# Clinical MR imaging in Parkinson’s disease: How useful is the swallow tail sign?

**DOI:** 10.1002/brb3.2202

**Published:** 2021-05-24

**Authors:** Jannik Prasuhn, Alexander Neumann, Robert Strautz, Shalida Dreischmeier, Felicitas Lemmer, Henrike Hanssen, Marcus Heldmann, Peter Schramm, Norbert Brüggemann

**Affiliations:** ^1^ Institute of Neurogenetics University of Lübeck Lübeck Germany; ^2^ Department of Neurology University Medical Center Schleswig‐Holstein Lübeck Germany; ^3^ Center for Brain, Behavior, and Metabolism University of Lübeck Lübeck Germany; ^4^ Department of Neuroradiology University Medical Center Schleswig‐Holstein Lübeck Germany; ^5^ Department of Psychiatry and Psychotherapy University Medical Center Schleswig‐Holstein Lübeck Germany; ^6^ Institute of Psychology II University Medical Center Schleswig‐Holstein Lübeck Germany

**Keywords:** Parkinson's disease, Neuroimaging, SWI, susceptibility‐weighted imaging, swallow tail sign

## Abstract

**Background:**

With conventional MRI, no Parkinson's disease (PD)‐specific abnormalities can be detected. However, there is a critical need for accompanying neuroimaging markers to guide the diagnosis. With high‐resolution susceptibility‐weighted MRI (SWI) sequences, the imaging of nigrosome‐1 (N1) is possible. The so‐called swallow tail sign (STS) has been proposed as a suitable neuroimaging marker for the diagnosis of PD.

**Objectives:**

To investigate whether the absence of the STS can be applied for distinguishing PD patients from healthy controls (HCs).

**Methods:**

SWI images of 44 PD patients and 50 age‐ and gender‐matched HCs were investigated using a 3T MRI scanner. Two trained neuroradiologists blind‐rated the images and evaluated whether the STS was absent (1) on one side or (2) both sides of the participant's midbrain.

**Results:**

Our results confirmed good interrater reliability comparable to previously published studies. However, we did not identify any group differences between PD patients and HCs. Measures of diagnostic values revealed overall poor diagnostic performance.

**Conclusions:**

Even though previously stated, our study does not confirm the potential use of the STS as a supportive neuroimaging marker for PD in a clinical setting. In conclusion, there is a critical need for improvements in N1‐targeted MRI sequences and the development of advanced segmentation algorithms.

## Graphical abstract

Examples for a healthy‐appearing swallow tail sign in a diseased individual (A) and an absent swallow tail sign in a healthy individual (B).

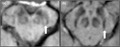

## 1 INTRODUCTION

Structural magnetic resonance imaging (MRI) in Parkinson's disease (PD) patients is applied in clinical practice to rule out potential differential diagnoses. Thus far, no reliable and widely accepted PD‐specific MRI marker is available. However, there is a critical need for accompanying neuroimaging markers to guide the diagnosis of PD given the diagnostic uncertainty at an early stage. Recent publications suggest that the swallow tail sign (STS) as assessed by susceptibility‐weighted imaging (SWI) can play a role in the diagnosis of PD and the differential of other parkinsonian syndromes (Meijer et al., 2016). In this context, high‐resolution SWI MRI sequences allow the imaging of nigrosome‐1 (N1), one of the neuroanatomical key structures in PD pathogenesis. N1 contains dopaminergic cells of the substantia nigra (SN) pars compacta (SNpc) and plays a crucial role in the underlying pathophysiology of PD (Schmidt et al., 2017). In healthy individuals, the hyperintense linear‐shaped N1 is located in the posterior third of the SN and surrounded by hypointense areas at the frontal and lateral portions. This configuration is reminiscent of a swallow tail (Schwarz et al., 2014). The absence of the swallow tail is considered to be characteristic to PD, and its potential significance has been described in a systematic review and meta‐analysis (Mahlknecht et al., 2017). Although current evidence suggests that this novel MRI feature may be a suitable marker for PD, acquisition techniques, for example, differences in magnetic field strength are still heterogeneous and results inconclusive (Schmidt et al., 2017). Here we aimed to determine the accuracy of image evaluation in a clinical setup. Even though some published studies already evaluated the diagnostic significance of the STS and addressed the aforementioned research question, we performed a reproducibility study to assess the potential use of the STS in our cohorts.

## 2 MATERIALS AND METHODS

### 2.1 Demographics and clinical examination

All study participants gave written informed consent in accordance with the revised version of the Declaration of Helsinki before enrollment. The ethics committee of the University of Lübeck gave their formal approval before the start of this study. Forty‐four PD patients (± standard deviation) (female/male: 20/24; mean age: 67.8 ± 8.4 years) and 50 age‐ and gender‐matched healthy controls (HC; female/male: 22/28; mean age: 70.8 ± 6.3 years) were enrolled in this study. All study participants underwent a thorough interview concerning their medical history and a clinical examination following the MDS‐UPDRS protocol. The clinical diagnosis for all PD patients was determined by movement disorders specialists in accordance with the MDS clinical diagnostic criteria for clinically established (as the highest level of diagnostic certainty) PD (Postuma et al., 2015). This assessment also included the thoroughful exclusion of absolute exclusion criteria (e.g., supranuclear gaze palsy), the absence of *red flags* (e.g., the rapid progression of gait impairment or requiring regular use of wheelchair within 5 years of disease onset), and the presence of at least two supportive diagnostic criteria (e.g., a clear beneficial response to dopaminergic therapy, which was present in all investigated individuals of the disease group).

### 2.2 MRI acquisition and analysis

Structural MR imaging was performed at the CBBM Core Facility Magnetic Resonance Imaging of the University of Lübeck using a 3‐T Siemens Magnetom Skyra scanner equipped with a 64‐channel head‐coil. For SWI, a 2D gradient echo (GRE) sequence with the following acquisition parameters has been acquired: TR=27 ms; TE=20 ms; MTC off; SWI on; flip angle 15°; 1x1x2 mm^3^ resolution; 120 × 220×220 mm^3^ field of view; acquisition time 4.54 min, transversal orientation, phase‐encoding direction P»A. The repeated measurements were coregistered before averaging. The chosen MRI protocol parameters adhere to a standard and widely available SW imaging protocol as often used in clinical practice (e.g., for evaluating microbleeds). We employed this approach to ensure the generalizability of our findings in a clinical setting. T1 imaging: Additionally, structural images of the whole brain using a 3D T1‐weighted MP‐RAGE sequence were acquired (TR=1900 ms; TE=2.44 ms; TI=900 ms; flip angle 9°; 1 × 1×1 mm^3^ resolution; 92 × 256×256 mm^3^ field of view; acquisition time 4.33 min, sagittal orientation, phase‐encoding direction A»P) and evaluated by a trained neuroradiologist to rule out the presence of conflicting structural lesions or relevant comorbidities (e.g., normal pressure hydrocephalus or vascular parkinsonism). Two trained neuroradiologists (A.N., P.S.) blind‐rated the SWI images and evaluated whether STS was absent on one (1) or both sides (2) of the participant's midbrain. Both raters had access to all axial slices for subsequent assessment of the STS. The accuracy of the classification was assessed against the clinical diagnosis of PD as the golden standard.

### 2.3 Statistics

Interrater reliability (IRR) was assessed with Cohen's Kappa statistic; afterward, a consensus agreement following a personal discussion of both raters was reached, upon disagreement. Chi‐square tests (*X^2^*) were employed to evaluate group differences regarding the one or two‐sided absence of the STS in PD patients compared to HCs. Contingency tables were calculated for measures of diagnostic value (e.g., sensitivity; see Table 1). In addition, the area under the receiver‐operator characteristics curve (ROC‐AUC) was calculated for both conditions to assess the overall diagnostic performance. All statistical tests were performed as implemented in SPSS 26 (IBM Corp. Released 2017. IBM SPSS Statistics for Macintosh, Version 25.0. Armonk, NY: IBM Corp.).

**TABLE 1:** Summary of the diagnostic performance metrics in both conditions following previous rater consensus agreement

Diagnostic parameters	Unilateral absence of the STS	Bilateral absence of the STS
Sensitivity	37%	9%
Specificity	78%	94%
PPV	61%	57%
NPV	59%	54%
False positives	39%	43%
False negatives	41%	54%
ROC‐AUC	0.583	0.515

Note

In general, the absence of the STS tends to be more specific than sensitive. However, the diagnostic value is negligible concerning the high false‐positive and false‐negative rates. The low diagnostic performance is summarized in the ROC‐AUCs values, which did not significantly outperform pure chance.

Abbreviations: NPV, negative predictive value; PPV, positive predictive value; ROC‐AUC, receiver‐operator characteristics area under the curve; STS, swallow tail sign.

## 3 RESULTS

### 3.1 Demographics and clinical data

The PD patients showed mean values (± standard deviation) for the disease duration of 7.4 ± 5.0 years, the MDS‐UPDRS‐I score of 11.0 ± 6.0, the MDS‐UPDRS‐II score of 11.0 ± 7.4, the MDS‐UPDRS‐III score of 27.3 ± 10.1, the MDS‐UPDRS‐IV score 4.1 ± 4.0, and the total MDS‐UPDRS score of 53.5 ± 19.6. The mean Hoehn & Yahr scale was 1.7 ± 1.1, and the Levodopa Equivalent Dose (LEDD) was 667 ± 386 mg/d.

### 3.2 Our study showed reasonable IRR for the assessment of the STS

Kappa statistics for the unilateral absence of the STS showed substantial (κ = 0.67, *p* >.001) and for the bilateral absence of the STS moderate (κ = 0.59, *p* >.001) interrater agreement according to the classification of Landis and Koch (Landis & Koch, 1977).

### 3.3 No significant group differences could be observed

No group differences were present between PD patients and HCs, neither for the unilateral absence of the STS (*X* ^2^(1)=3.097, *p*=.078, *n* = 94) nor for the bilateral absence of the STS (*X* ^2^(1,)=0.324, *p*=.569, *n* = 94). Measurements of diagnostic value (e.g., sensitivity) are presented in the table. In summary, none of the diagnostic metrics provide any benefit for clinical decision support, which is additionally highlighted by the poor ROC‐AUCs (see Table 1).

## 4 DISCUSSION

**FIGURE 1:**
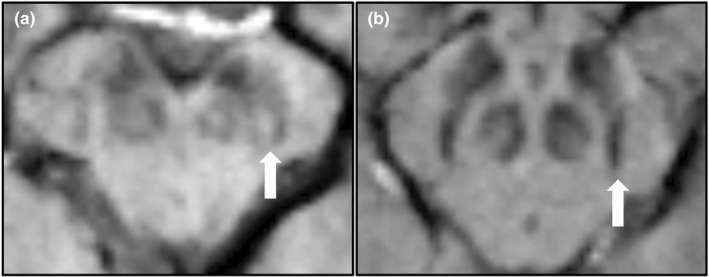
Examples for a healthy‐appearing swallow tail sign (STS) in a diseased individual (a) and an absent STS in a healthy individual (b). Shown here are axial midbrain slices mapped via susceptibility‐weighted imaging (SWI). White arrows are indicating the different configuration of Nigrosome‐1 (N1)

In summary, our study is methodologically well in line with previously published reports. Our IRR illustrates that the overall detection of the STS was robust and provides general comparability to other already published studies (Rizzo et al., 2019). The unilateral absence of the STS may be more suitable for further studies highlighted by the overall better IRR and diagnostic performance metrics with a trend toward a difference between PD and HCs. However, we were not able to demonstrate that there is unequivocal diagnostic value to identify PD patients, which stands in clear contrast to previous reports (Mahlknecht et al., 2017). Our study included a comparable number of cases, indicating that the lack of relevant group differences is not driven by the lack of sufficient statistical power (Bae et al., 2016; Sung et al., 2016). However, the number of subjects per group was unbalanced in some previous studies, which may skew the diagnostic performance toward falsely better sensitivity and positive predictive values (Bae et al., 2016). Our SWI sequence is well in line with former studies performed on 3T MRI scanners and is routinely applied in clinical practice. The post‐mortem study of Kau et al. (Kau et al., 2019) identified microvessels within the SNpc as a potential confounder for SWI measurements: In eight out of nine HCs, one or more microvessels were detected medial to the STS or at least unilaterally in the medial part of the STS formation. Intrinsic vessels of the midbrain dopaminergic system may occasionally be responsible for false‐positive identification of the masked STS in the assessment of the dorsolateral SNpc (Figure 1). Therefore, both iron deposits and microvessels might contribute to the hypointense signal surrounding N1 in the SWI of normal aged midbrains without being specific to PD (Postuma et al., 2015). However, anatomical variation is unlikely to be present only in our study and, therefore, cannot cause these conflicting results solely. In another study, Oustwani et al. found that STS was absent in 21% of HCs, implicating a considerable number of false‐positive ratings in HCs (Oustwani et al., 2017), which is even more pronounced in our study (see Table 1). Additionally, there were no differences in the presence of the STS between PD patients and other parkinsonian syndromes, which puts the pathophysiology‐orientated interpretation of the STS in PD into perspective (Oustwani et al., 2017). This argument is also underlined by the study of Shams et al., where the usefulness of the STS in patients with Lewy Body Dementia (LBD) was examined (Shams et al., 2017). Even though many previous studies confirmed severely abnormal DaTscan imaging in patients with LBD (Papathanasiou et al., 2012), as a marker of the direct loss of dopaminergic neurons in the SNpc, the absent STS could only be detected in 63% of the diseased cases (Shams et al., 2017). Another study comparing the diagnostic value of the STS in distinguishing LBD from Alzheimer's disease patients showed similar results (Oliveira et al., 2017). In almost all previous publications, occasional failure of the STS was accepted and interpreted as a lack of diagnostic value for the differential diagnosis of PD and other parkinsonian syndromes (Mahlknecht et al., 2017). As of now, the visualization of the STS cannot be easily achieved in a clinical setting using a standardized MRI protocol where the STS interpretation is comparable between different centers and well and less experienced neuroradiologists. Previous reports state that, for example, by increasing the static magnetic field strength or employing a more sophisticated MRI sequence design, better image contrasts and resolutions can be reached (Lee et al., 2020). However, these approaches are currently not available for standard clinical assessment of patients. Based on the long prodromal phase and the disease course of PD patients, longitudinal scans would be desirable to reevaluate the diagnostic value of the STS at distinct time points. One attempt is to follow‐up subjects at risk (e.g., subjects with isolated REM sleep behavior disorder or asymptomatic carriers of mutations in PD‐related genes) who show a clear neurodegeneration in the presynaptic dopaminergic innervation as assessed by, for example, DaTSCANs. Whether the absence or the presence of a dichotomous radiological sign (like the STS) can, in general, be used for the diagnosis of PD patients and maps one's individual disease course is, however, questionable. Most likely, the standardized analysis of the shape, volume, or intensity of the SN (e.g., by including different imaging modalities) will yield a substantially higher diagnostic value in the horizontal and longitudinal assessment of PD patients. However, these approaches have not yet been sufficiently evaluated in clinical practice. As our study fails to distinguish PD patients from HCs, the diagnostic use of the STS in the differential diagnoses of other parkinsonian syndromes should be discussed cautiously in a clinical setting. To further improve the diagnostic value of the STS, optimized imaging sequences and techniques are to be applied. One limitation of our study is that these advanced MRI acquisition schemes were not applied here. However, the use of ultra‐high field MRI (i.e., utilizing 7 T MRI scanner), even though desirable, is still no essential part of the clinical routine and, therefore, might not translate into clinical practice in the near future (Ladd et al., 2018). In this context, it is crucial to consider that diagnostic imaging in a clinical setting is also substantially hindered by the overall image acquisition time and the availability of high spatial‐resolution MRI sequences (which often lack formal approval by the respective authorities).

## 5 CONCLUSION

Even though previously highlighted, our study does not provide evidence for the diagnostic use of the STS as a supportive neuroimaging marker for manifest PD. Our study, therefore, contradicts the conclusion of a published systematic review, meta‐analysis, and earlier peer‐reviewed studies. As our study is well in line with the methodology of previous studies, the contradictory results might point toward a potential publication bias and should raise concerns about the diagnostic value of the STS in PD patients. Whether improvements of N1‐targeted MRI sequences and the development of advanced segmentation algorithms will improve the significance of the STS in PD needs to be critically evaluated.

## CONFLICT OF INTEREST

The authors have no conflict of interest to report.

### PEER REVIEW

The peer review history for this article is available at https://publons.com/publon/10.1002/brb3.2202.
